# Efficient Photothermal Elimination of Formaldehyde under Visible Light at Room Temperature by a MnO*_x_*-Modified Multi-Porous Carbon Sphere

**DOI:** 10.3390/ma15134484

**Published:** 2022-06-25

**Authors:** Wanpeng Liu, Liu Shi, Rongyang Yin, Pengfei Sun, Jinming Ren, Yongming Wang

**Affiliations:** 1 Power China Huadong Engineering Corporation, Hangzhou 311122, China; liu_wp@hdec.com (W.L.); ren_jm@hdec.com (J.R.); 2 Department of Chemistry, Key Laboratory of Surface & Interface Science of Polymer Materials of Zhejiang Province, Zhejiang Sci-Tech University, Hangzhou 310018, China; sl996158058@163.com (L.S.); yinrongyang0220@163.com (R.Y.); sunpf@zju.edu.cn (P.S.)

**Keywords:** photothermal catalytic, MnO*_x_*, multi-porous carbon sphere, formaldehyde, indoor air pollution

## Abstract

Volatile organic compounds (VOCs) exert a serious impact on the environment and human health. The development of new technologies for the elimination of VOCs, especially those from non-industrial emission sources, such as indoor air pollution and other low-concentration VOCs exhaust gases, is essential for improving environmental quality and human health. In this study, a monolithic photothermocatalyst was prepared by stabilizing manganese oxide on multi-porous carbon spheres to facilitate the elimination of formaldehyde (HCHO). This catalyst exhibited excellent photothermal synergistic performance. Therefore, by harvesting only visible light, the catalyst could spontaneously heat up its surface to achieve a thermal catalytic oxidation state suitable for eliminating HCHO. We found that the surface temperature of the catalyst could reach to up 93.8 °C under visible light, achieving an 87.5% HCHO removal efficiency when the initial concentration of HCHO was 160 ppm. The microporous structure on the surface of the carbon spheres not only increased the specific surface area and loading capacity of manganese oxide but also increased their photothermal efficiency, allowing them to reach a temperature high enough for MnO*_x_* to overcome the activation energy required for HCHO oxidation. The relevant catalyst characteristics were analyzed using XRD, measurement of BET surface area, scanning electron microscopy, HR-TEM, XPS, and DRS. Results obtained from a cyclic performance test indicated high stability and potential application of the MnO*_x_*-modified multi-porous carbon sphere.

## 1. Introduction

Volatile organic compounds (VOCs) have been widely associated with ecological damage and human health problems [1]. For the removal of different types of VOCs, such as those emitted through industrial production processes, waste incineration, and medical waste, current technologies use methods such as catalytic oxidation, biological drip filtration, or even direct combustion [2,3]. These methods effectively reduce VOC emissions, thereby suppressing environmental pollution. However, these methods demand large technical input and management costs, making them unsuitable for scenarios such as indoor VOCs air pollution or when low concentrations of VOCs are emitted by small businesses. In view of this, new VOC-removal methods have been developed, such as photocatalysis, which uses photocatalytic materials to generate excited electrons under visible or ultraviolet light, resulting in the oxidative decomposition of VOC molecules at the catalyst surface [4]. Through constantly improving the performance of photocatalysts, this method is being increasingly recognized in the field of VOC purification.

In recent years, synergistic photothermocatalysis has been considered an efficient and low-carbon technology for the removal of VOCs. This technology has the advantages of both photocatalysis and thermocatalysis, which enables the catalyst to be activated by a natural light source without the need for extra energy and stimulates the rising surface temperature of the catalyst to the level required for VOC oxidation. Transition metal oxides (TMOs) are always selected as the main active ingredients for photothermocatalytic materials because their relatively large d-orbital splitting energy absorbs UV-vis and partial infrared light, resulting in an efficient photothermal conversion [5,6]. The variable valence states and active redox properties of TMOs are also favorable for the oxidative removal of low-concentration VOCs [7]. In addition, black carbonaceous materials have been introduced, which can cause efficient absorption of near-infrared wavelengths and a rapid increase in the temperature of the reaction surface, thereby enabling modified TMOs to overcome the activation energy required for VOC oxidation [8,9].

In this study, multi-porous carbon spheres were prepared using available glucose, which was then used to load stable manganese oxide impregnated with potassium permanganate (KMnO_4_) solution, forming typical photothermal catalysts. Formaldehyde (HCHO), one of the most common indoor VOC pollutants, was selected as a probe for testing the performance of the photothermal catalysts. We found that the surface temperature of the catalysts could reach up to 93.8 °C and achieved a maximum HCHO removal efficiency of 87.5% under visible light only. The surface properties of the catalysts were characterized using X-ray diffraction (XRD), Brunauer-Emmett-Teller (BET) surface area, scanning electron microscopy (SEM), HR-TEM, X-ray photoelectron spectroscopy (XPS), and UV-Vis-NIR diffuse-reflectance spectroscopy (DRS), whereas results obtained from the cyclic performance showed high stability of the catalysts, implying a high application potential.

## 2. Materials and Methods

### 2.1. Catalyst Synthesis

Commercially available glucose (C_6_H_12_O_6_) was used for the experiments. An aliquot of 4.0 g glucose was dissolved in 40 mL distilled water, transferred into a Teflon-lined stainless-steel autoclave, and maintained at 180 °C for 10 h. After cooling down to room temperature, the brown powder was obtained by filtration, which was then washed repeatedly with deionized water and finally dried at 80 °C under a vacuum. The obtained sample was named CNS.

The prepared CNS was then immersed into a ZnCl_2_ solution and stirred for 6 h. Thereafter, the powder was dried at 120 °C and treated at 400 °C, 500 °C, and 600 °C under nitrogen for 2 h each. These treated powders were then washed in 0.5 M HCl and distilled water alternatively to remove a zinc oxide and dried at 80 °C under a vacuum. Based on the treated temperature, these counterpart samples were denoted as PCNS-400, PCNS-500, and PCNS-600.

The Mn-modified CNS and PCNS samples were prepared by immersing them in KMnO_4_ solutions with different concentrations, and the catalysts obtained were labeled as xMnC-y and xMnC-y-z. For example, the catalyst named 0.05MnC-30 was prepared by immersing CNS into 0.05 mol·L^−1^ of a KMnO_4_ solution and stirred for 30 min; the catalyst named 0.05MnC-30-500 was prepared by immersing PCNS-500 into 0.05 mol·L^−1^ of a KMnO_4_ solution and stirred for 30 min. All KMnO_4_-treated samples were dried at 60 °C for 5 h before being used for HCHO removal.

### 2.2. Characterizations

XRD with Cu Kα radiation was used to characterize the crystal structure on a DX-2700 diffractometer (Dandong Haoyuan Instrument Co. Ltd., Dandong, China). Morphology and microstructure analyses were conducted on a scanning electron microscope (HITACHI SU8100, Tokyo, Japan) equipped with an energy-dispersive X-ray spectrometer. Transmission electron microscopy (TEM) images were obtained using transmission electron microscopy (TEM, JEOL JEM-2100, Tokyo, Japan).

The BET surface area was determined using N_2_ physisorption at 77 K, Micromeritics ASAP 2020 surface area, and a porosity analyzer. Catalyst degassing pre-treatment was conducted at 150 °C for 2 h under a vacuum. The surface area was calculated from the N_2_ isotherm data using the BET model [10]. Micropore volume and micropore diameter were measured using t-plot analyses [11].

XPS measurements were conducted using a Thermo (Waltham, MA, USA) ESCALAB 250 spectrometer with Al Kα X-ray (*h_ν_* = 1486.6 eV) radiation as the excitation source. Charging of the catalysts was calibrated by setting the binding energy (BE) of adventitious carbon (C1s) to 284.6 eV.

UV-Vis-NIR diffuse-reflectance spectra were analyzed on a spectrophotometer (U-4100, HITACHI, Tokyo, Japan). A certain quality of catalyst powder is pressed in the specific grinding tool provided by this spectrophotometer to obtain the same thickness as the tested sample.

The surface temperature increases of the different catalysts under a xenon lamp irradiation were detected by an AT-600 infrared thermometer (Smart Sensor, Dongguan, China). All sample powder was daubed on a glass sheet and was put into the reactor to receive light irradiation.

### 2.3. Catalytic Tests for HCHO Removal

The photothermal catalytic reaction was carried out using a 300 W xenon lamp (HSX-F300, Beijing NBet, Beijing, China) as a light source, and the HCHO removal activity of different samples was obtained using a 500 mL gas-phase reactor. First, the sample was placed in the center of the gas-phase reactor. After 2.0 μL of HCHO (37%) solution was injected into the reactor, we preheated the gas-phase reactor for less than 10 °C to make the HCHO solution completely evaporate to a stable 160 mg/L gas state. The xenon lamp was turned on to initiate the photothermal catalytic reaction. The HCHO removal activity of the catalyst was indirectly evaluated by measuring absorbance, which was obtained using phenol reagent spectrophotometry. The concentration change of HCHO in the reactor was analyzed using a gas chromatograph (Agilent 6890, Santa Clara, CA, USA) equipped with a flame ionization detector. For the catalyst cycle test, the used catalyst was placed in a transparent drying oven for 24 h before the next round of experiments.

## 3. Result and Discussion

### 3.1. HCHO Removal Efficiency

**Figure 1:**
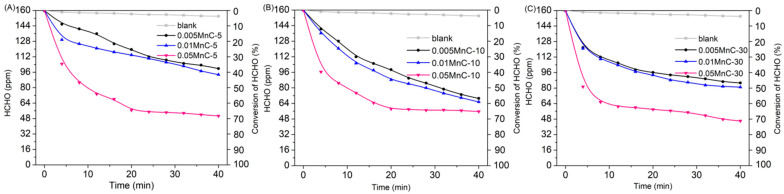
The HCHO elimination rate under xenon light irradiation by Mn-modified CNS catalysts with different synthesis conditions: (**A**) 0.005, 0.01, and 0.05 mol/L of potassium permanganate impregnated for 5 min; (**B**) 0.005, 0.01, and 0.05 mol/L of potassium permanganate impregnated for 10 min; and (**C**) 0.005, 0.01, and 0.05 mol/L of potassium permanganate impregnated for 30 min.

Impregnation times were used to test HCHO removal efficiency. As shown in Figure 1, the removal efficiency of HCHO can reach up to ca.75% within 40 min when KMnO_4_ concentration is 0.05 mol/L during the catalyst synthesis process. However, an extension of impregnation time during the catalyst synthesis process would, to some extent, improve the HCHO removal performance. This is especially the case when, as shown in Figure 1C, the removal efficiency of HCHO had reached ca.71.6% within the first 10 min, compared to the situations depicted in Figure 1A,B. The surface carbon of CNS reduces KMnO_4_ to produce loaded MnO*_x_*, causing the oxidation of HCHO [12], and KMnO_4_ concentration and immersion time determine the quality of the MnO*_x_* attached to the CNS surface. According to the above orthogonal test results, a synthesis condition of 0.05 mol/L of KMnO_4_ impregnated for 30 min can achieve a satisfactory HCHO removal rate.

**Figure 2:**
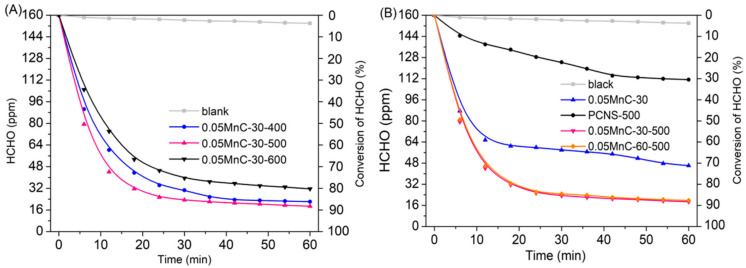
(**A**) The HCHO elimination rate under xenon light irradiation by Mn-PCNS catalysts under processing temperatures. (**B**) The HCHO elimination rate under xenon light irradiation by 0.05MnC-30, PCNS-500, 0.05MnC-30-500, and 0.05MnC-60-500 catalysts.

**Figure 3:**
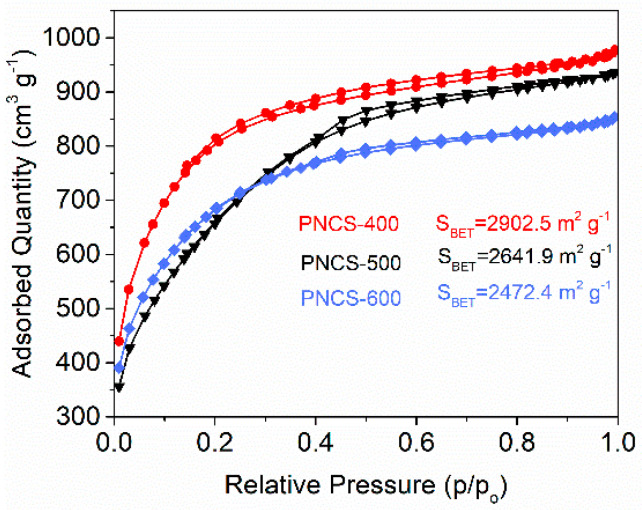
N_2_ adsorption (full symbols)–desorption (empty symbols) isotherms of PCNS-400, PCNS-500, and PCNS-600.

Figure 2A shows the removal efficiency of HCHO by the Mn-PCNS catalyst under different PCNS-treated temperatures. We found that the catalyst synthesized by PCNS-500 with 0.05 mol/L of KMnO_4_ had the best HCHO removal efficiency, primarily due to it having a more optimized pore structure and HCHO adsorption capacity. As shown in Figure 3 and Table 1, the nitrogen-sorption isotherms of PCNS-500 are type IV mesoporous materials and the average pore diameter (nm) of PCNS-500 is clearly wider than those of PCNS-400 and PCNS-600. For PCNS-600, the two isotherms almost coincide, indicating that the pore structure effect is poor, which is probably due to the sintering shrinkage of the surface caused by too high a roasting temperature. The isotherms of PCNS-400 are also different from PCNS-500, which exhibits a typical curve of activated carbon type with narrow crack pores, and have not presented a typical mesoporous hysteresis loop [13,14]. Figure 2B shows a comparison of HCHO elimination rate under xenon light irradiation between 0.05MnC-30, PCNS-500, and 0.05MnC-30-500. The 0.05MnC-30-500 achieved a HCHO removal rate of 87.5%, which increased by ca.25% in 0.05MnC-30. The 0.05MnC-60-500 catalyst was also synthesized to investigate the influence of an impregnation time of up to 60 min on the performance of the catalyst, and the result did not show any significant difference between the 0.05MnC-60-500 and 0.05MnC-30-500 catalysts.

### 3.2. Influence of Light Irradiation

**Figure 4:**
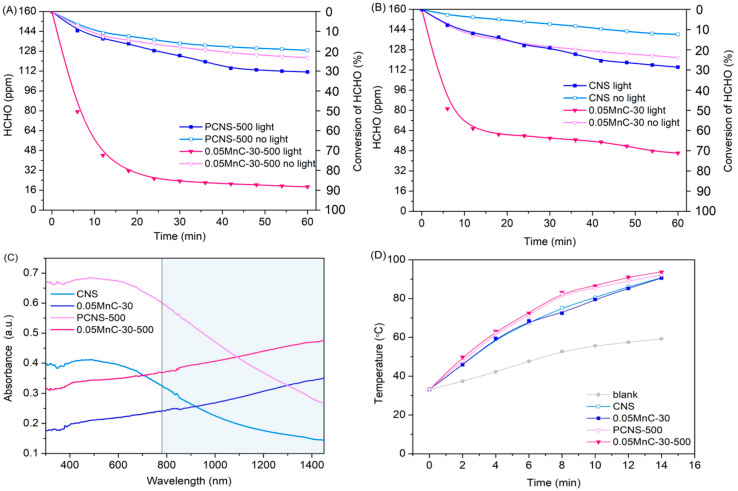
(**A**) Catalytic performance of PCNS and Mn-PCNS for HCHO elimination with and without irradiation. (**B**) Catalytic performance of CNS and Mn-CNS for HCHO elimination with and without irradiation; (**C**)UV-Vis-NIR DRS profiles of CNS, 0.05MnC-30, PCNS-500, and 0.05MnC-30-500. (**D**) Change in surface temperature of catalysts under visible light irradiation.

Figure 4A,B show that the activity of the catalysts is optimal under light. In the absence of light, the HCHO removal performance of catalysts Mn-PCNS and Mn-CNS is less than 20%, which is primarily due to the adsorption of the materials themselves. Figure 4C shows the UV-Vis-NIR DRS test of CNS, 0.05MnC-30, PCNS-500, and 0.05MnC-30-500. We found that the near-infrared (wavelength region > 780 nm) absorption was greater in the PCNS catalysts than in the CNS catalysts. As is known, the surface temperature of materials can rise by absorption of near-infrared light [15]. In Figure 4D, the surface temperature of the PCNS catalyst is clearly higher than those of the CNS catalysts, and the maximum of 93.8 °C can be reached for the 0.05MnC-30-500 catalyst after close contact illumination of xenon lamp light for 14 min. Thus, it was concluded that the optimized mesoporous structure of Mn-PCNS catalysts promoted the absorption of near-infrared light to a greater degree than CNS, which promoted an increase in the surface temperature and the Mars-van Krevelen mechanism of MnO*_x_* for the HCHO oxidation [16].

### 3.3. XRD Analysis

**Figure 5:**
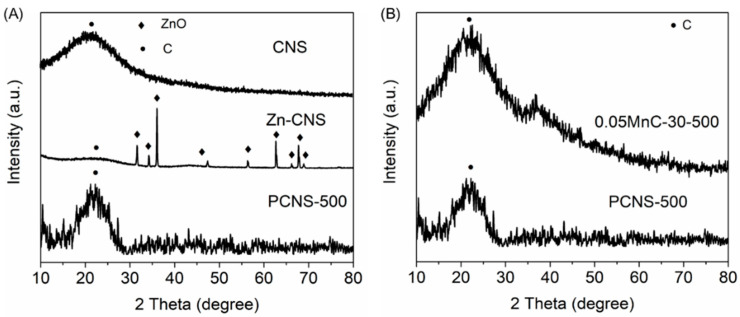
(**A**) XRD profiles of CNS, Zn-CNS, and PCNS-500; (**B**) XRD profiles of 0.05MnC-30-500 and PCNS-500.

Figure 5 shows the XRD profiles of CNS, PCNS-500, and 0.05MnC-30-500. As shown in Figure 5A,B, the characteristic peak at approximately 22.5° is ascribed to carbon for CNS, PCNS-500, and 0.05MnC-30-500. After being treated with ZnCl_2_ and calcined at 500 °C, the Zn-CNS sample without that had no acid washing developed a hexagonal ZnO phase at 2 theta = 30–70° [17], which implies that the activation process of CNS converted ZnCl_2_ into ZnO. The Zn-CNS sample was then washed using 0.5 M of HCl, and the obtained sample (PCNS-500) had only carbon, indicating that the ZnO formed at the surface was removed, leaving a porous structure. Figure 5B shows an akhtenskite phase (2 theta = 36.5°) for 0.05MnC-30-500 [18,19], which indicates the stable formation of MnO*_x_* at the surface of the carbon spheres with porous structures after being immersed in 0.05 mol/L of KMnO_4_ solution.

### 3.4. Morphological Characteristics

**Figure 6:**
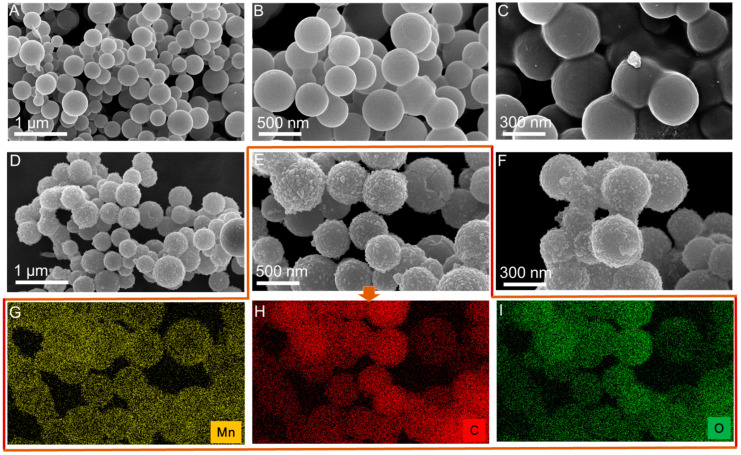
SEM images of PCNS-500 (**A**–**C**), SEM images of Mn modified PCNS-5000 with 0.05 mol/L of potassium permanganate impregnated for 30 min (**D**–**F**), EDX-mapping test of element Mn, C, and O of Mn modified PCNS-500 ((**G**–**I**) related to SEM image of (**E**)).

**Figure 7:**
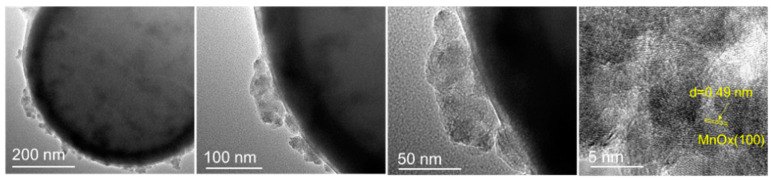
HR-TEM profile of sample 0.05MnC-30-500.

The morphologies of PCNS-500 and 0.05MnC-30-500 were also characterized using SEM, as shown in Figure 6A–F, where the PCNS-500 and 0.05MnC-30-500 both exhibit optimal ball shapes in the size range of 300–500 nm. For the 0.05MnC-30-500 sample, the surface of the carbon sphere showed a certain burr and concave-convex feeling, which was due to the formation of MnO*_x_* on the surface of the carbon sphere. The HR-TEM of the 0.05MnC-30-500 catalyst (see Figure 7), which has a d-spacing of 0.49 nm, matching the interlayer distance of (100) facet of MnO*_x_* crystal, also proves this [18,19].

### 3.5. Surface Properties

**Figure 8:**
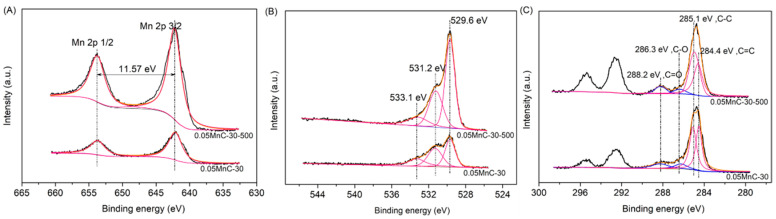
XPS spectrum of 0.05Mn-C-30-500 and 0.05MnC-30: (**A**) Mn2p; (**B**) O1s: (**C**) C1s.

XPS analyses of the 0.05Mn-C-30-500 and 0.05MnC-30 samples were conducted to identify the states of the surface chemical elements. As shown in Figure 8A, the BEs at 642.1 and 653.1 eV were ascribed to Mn 2p3/2 and Mn 2p1/2 spin-orbit peaks for MnO_2_ [20,21]. The energy separation between Mn 2p3/2 and Mn 2p1/2 was 11.57 eV, which illustrates that Mn has a +4 valency. In Figure 8B, the O 1s spectra of 0.05Mn-C-30-500 and 0.05C-30 can be separated into three peaks at 529.6, 531.2, and 533.1 eV, respectively. The peak at 533.1 eV can be ascribed to carbon-oxygen bonds (O_C-O_) [22], the peak at 532.1 eV can be ascribed to surface reactive oxygen species (O_sur_) [23,24], and the peak at 533.1 eV can be ascribed to lattice oxygen (O_lat_) [21,25]. According to the Mn2p spectra, the peak intensity of 0.05Mn-C-30-500 is much stronger than that of 0.05MnC-30, which indicates that more manganese oxide will be doped on the surface of the porous carbon sphere. The increased MnO_2_ formed will cause an increase in the content of O_sur_ and O_lat_, the former having interacted with the Mn atom via a covalent coordination bond or a hydrogen bond, which will be the key oxygen species involved in the surface oxidation of HCHO [21,26]. The surface lattice oxygen will also induce the Mars-van Krevelen (MVK) mechanism for the oxidation of C-H with an increase in reaction temperature [20,21]. It can also be seen from Table 1 that the percentage of O_sur_ on the surface of 0.05Mn-C-30-500 is 34.2%, which is higher than that of 0.05MnC-30, indicating that 0.05Mn-C-30-500 has a better HCHO removal performance.

In Figure 8C, the C1s spectra of the 0.05Mn-C-30-500 and 0.05MnC-30 can be separated into four main components at 284.4, 285.1, 286.3, and 288.2 eV, respectively, which are assigned to sp^2^-hybridized graphite-like C=C bonding [27], sp^3^ C-C [28], C-O [29], and O-C=O groups [30]. The oxygen-rich groups of C-O and O-C=O at the surface of the carbon spheres are also produced by doped MnO_2_, proving that an increase in MnO_2_ content results in an increase in the C-O and O-C=O contents. The peaks at 295.3 eV and 292.7 eV in Figure 8C are characteristic of potassium oxide [31], which is generated by the residual K^+^ of used KMnO_4_.

### 3.6. Catalyst Recycling Performance

**Figure 9:**
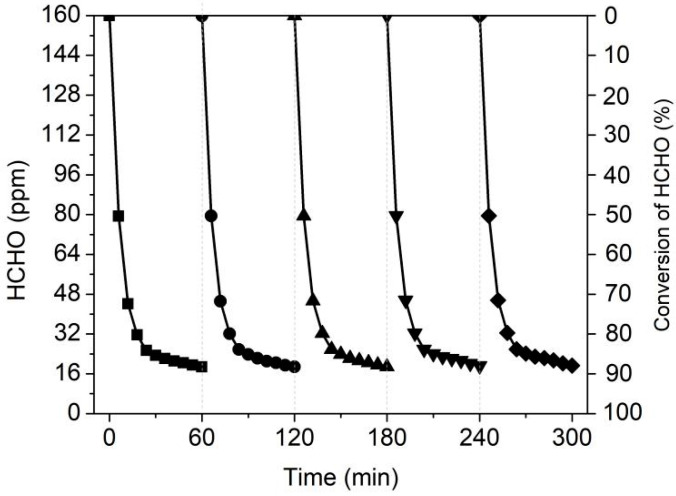
Cyclic performance test of 0.05MnC-30-500 for HCHO removal.

The recycling performance of the 0.05MnC-30-500 sample was also tested to verify its application potential. As shown in Figure 9, this catalyst exhibits a very stable HCHO removal rate, as shown by five recycling tests conducted, and its HCHO removal efficiency always remains at ca. 87%. This result proved that the synthetic 0.05MnC-30-500 has extremely high stability under visible light irradiation, which shows great application potential in indoor HCHO elimination or related application scenario where removal of low concentration VOCs are desired.

## 4. Conclusions

In this work, a MnO*_x_*-modified multi-porous carbon sphere was synthesized by common glucose and was used for indoor formaldehyde purification. It was found the synthesized catalyst exhibits good photothermal conversion performance, which can caused the surface temperature of the reaction interface reach to 93.8 °C at maximum under xenon light illumination. The surface-modified MnO*_x_* exhibited good oxidation performance at this temperature and achieved an 87.5% HCHO removal efficiency due to abundant O_sur_ and surface O_lat_. BET analysis result demonstrated the surface mesoporous structure of synthetic carbon spheres by ZnCl_2_ treatment, which further increased the adsorption and removal efficiency of formaldehyde. This work is of great significance for the development of indoor VOC purification materials and the expansion of relevant literature.

## Figures and Tables

**Table 1:** Pore parameters of PCNS treated at different calcination temperature and XPS result of O1s for 0.05MnC-30-500 and 0.05MnC-30.

Pore Parameters				XPS			
Catalyst	BET Surface Area (m^2^/g)	Micropore Volume (cm^3^/g)	Average Pore Diameter (nm)	Catalyst	O1S		
					O_lat_	O_sur_ (%)	O_C-O_
PCNS-400	2902.5	0.34	1.59	0.05MnC-30-500	51.7	34.5	13.8
PCNS-500	2641.9	0.71	2.73	0.05MnC-30	50.4	32.5	17.1
PCNS-600	2472.4	0.16	1.46				

## Data Availability

Not applicable.
